# Supplementary material for: Thyme Essential Oil as a Potential Tool Against Common and Re-Emerging Foodborne Pathogens: Biocidal Effect on Bacterial Membrane Permeability
Source: Microorganisms. 2024 Dec 27;13(1):37. doi: 10.3390/microorganisms13010037 (PMC11768042; doi:10.3390/microorganisms13010037)
Supplement: Supplementary file 1 [file microorganisms-13-00037-s001.zip › microorganisms-3336617-supplementary.pdf]

microorganisms MDPI

Supplementary Materials

Table S1. Antimicrobial susceptibilities determined by VITEK® against S. enterica group B clinical isolate.

Antimicrobial Agent	*S. enterica* group B	
	[a]MIC	[b]Interpretation
Quinolones		[c]R
Amoxicillin	4	[d]S
Piperacillin	≤4	S
Cefotaxime	≤1	S
Ceftazidime	≤1	S
Cefepime	≤1	S
Ertapenem	≤0.5	S
Imipenem	≤0.25	S
Meropenem	≤0.25	S
Amikacin	≤2	S
Gentamicin	≤1	S
Tigecycline	≤0.5	S
Fosfomycin	≤16	S
Colistin	≤0.5	S
Trimetoprim	≤20	S

[a] MIC, minimum inhibitory concentration values of antibiotics; [b] Interpretation of the susceptibility test is based on the European Committee on Antimicrobial Susceptibility Testing (EUCAST) guidelines; [c] R, Resistant; [d] S, Sensitive.

Table S2. Antimicrobial susceptibilities determined by VITEK® against *Y. enterocolitca* and *L. monocytogenes* clinical isolates.

Antimicrobial agent	*Y. enterocolitica*		*L. monocytogenes*	
	[a]MIC	[b]Interpretation	MIC	Interpretation
Ampicillin	256	[c]R	0.75	[d]S
Benzylpenicillin	10	R	0.75	S
Ceftriaxone	0.25	S	[e]NT	NT
Ciprofloxacin	0.032	S	NT	NT
Eritromicin	5	S	0.25	S
Meropenem	0.032	S	0.125	S
Piperacillin	1.5	S	3	R
Tigecycline	0.25	S	NT	NT

[a]MIC, minimum inhibitory concentration values of antibiotics; [b]Interpretation of the susceptibility test is based on the European Committee on Antimicrobial Susceptibility Testing (EUCAST) guidelines; [c]R, Resistant; [d]S, Sensitive; [e]NT, Not Tested.
